# “*Say a Little but Say It Well*”: Assessing the Impact of Social Media Communication on Value Co-creation, Online Customer Experience, and Customer Well-Being

**DOI:** 10.3389/fpsyg.2022.901229

**Published:** 2022-07-05

**Authors:** Maheen Iqbal Awan, Amjad Shamim, Muhammad Shoaib Saleem

**Affiliations:** Management and Humanities Department, Universiti Teknologi PETRONAS, Seri Iskandar, Malaysia

**Keywords:** social media communication, value co-creation, cognitive experiential state, affective experiential state, online customer experience, customer wellbeing

## Abstract

The purpose of the study is to identify how both tourism service provider- and tourist-generated social media communication affect the value co-creation process and how this can affect online customer experience and customer wellbeing. A questionnaire survey was used and 361 valid responses were obtained from Malaysian citizens. The research findings showed that tourism service provider- and tourist- generated social media communication positively influence value co-creation. Similarly, value co-creation positively influences cognitive and affective experiential states and these two states positively influence customer wellbeing. Furthermore, value co-creation partially mediates the relationship between social media communication and online customer experience, whereas, online customer experiences also partially mediate the relationship between value co-creation and customer wellbeing. This study has tried to establish theoretical relationship between some significant variables and the findings would aid both academicians and practitioners in formulating strategies for future.

## Introduction

The importance of the tourism sector or tourism service providers (tourists destinations/attractions, hotels/resorts, restaurants, transportation, adventure and recreation, events/conferences/expo etc.) to the economic development of any country and its connected markets cannot be underestimated (Liu and Chou, 2016). As a result, destination-specific tourism and its management through tourism service providers continue to be very significant to present and potential tourism destinations (De Moya and Jain, 2013).

According to Godey et al. (2016), firms must make genuine efforts through effective and fruitful communication tactics to develop a positive perception of the tourism service provider. At the moment, businesses typically use Web 2.0 and other social network platforms for this purpose, which, due to their low cost and convenience, are well regarded by customers/tourists worldwide (Hudson et al., 2016). Tourists/customers, on the other hand, prefer social media for communication since they feel more empowered to give their negative or positive feedback (Eisingerich et al., 2014). It has also been observed that social media websites enable businesses to reach clients in an infinite number of ways and quantities, as well as help customers interact/participate in various activities (Mazzarol et al., 2007). Given the market’s evolution, strategies for efficiently managing both tourism service provider (TSP) and tourist-generated social media communication must be established (Huerta-Álvarez et al., 2020).

Furthermore, it is important to note that social media being a modern form of communication allows two-way communication. The tourism service providers communicate using audio and visual tools which in turn are assessed by the customers. The customers like, comment, share, or give feedback on the content shared by the tourism service provider. This two-way communication may lead to the value co-creation process where customers equally contribute in the process of value creation for the greater good (Casper Ferm and Thaichon, 2021). In addition, it is expected from the tourism service providers to assist customers with surfing, answering questions, and generating enjoyable and memorable online experiences, thus creating value for consumers and providing the reason tourists/customers stay loyal with a specific service provider (Kim et al., 2019).

Moreover, there are two elements to the online customer experience: cognitive experiential state (flow) and affective experiential state (Micu et al., 2019). It is critical to examine how these two states contribute to explaining total customer wellbeing when a customer/tourist experiences them as a result of value co-creation *via* social media communication. For most service providers, the results of consumption behaviors may be less apparent, and the bulk of customer experiences are likely to have both negative and positive effects on quality of life. However, little is known as to how tourism services affect the quality of life of consumers. It is necessary to evaluate the relevant service-related experiences that marketers must consider when designing services that create a good and significant difference in the wellbeing of their customers (Grzeskowiak and Sirgy, 2007).

The complexity of service systems is expanding with each passing day due to the engagement of and interactions with new actors. The tourism industry has also been greatly influenced by the rising complexity of service systems. This increase in complexity must be acknowledged and addressed through research (Baccarani and Cassia, 2017). Customer experience in online environments is one of the fields that is growing in importance as online technology and sales grow. Customers must interact, engage, contribute, and co-create on online platforms in order to have a positive experience (Bilgihan et al., 2014). Klaus (2014) proposed that the fundamental reasons underlying customer experience tactics that result in favorable or unfavorable customer experience must be investigated. In addition, Prebensen and Xie (2017) stressed the importance of conducting in-depth studies in experiential consumption contexts to improve the validity and reliability of the co-creation dimensions. Since social media provides a distinct platform for connection and communication, it may result in “distinctive and differentiated consumer experiences” (Pandey and Kumar, 2020). According to Lei et al. (2021), more study is needed to find effective stimuli (social media communication in our example) that can be created to allow value co-creation, generate positive customer experiences, and improve customer wellbeing. As per the authors’ information, scarce knowledge is available in the current body of knowledge about the effects of online customer experience that might condition the relationship between social media communication, value co-creation and customer wellbeing. Moreover, to elaborate on the theoretical link between social media communication, value co-creation, online customer experience, and customer wellbeing, we would be extending the Uses and Gratification theory and Horizontal Spill-over theory. Having said all that, in light of the both context and topic, the aim of this study is to identify how both tourism service provider- and tourist-generated social media communication affects the value co-creation process and how this can affect online experience and wellbeing of customers.

## Literature Review and Hypothesis Development

### Social Media Communication

In terms of business, the social web allows for faster access to a larger number of clients, allowing for ongoing engagement with the consumer base through active participation in social media channels (Mazzarol et al., 2007). Because social media allows customers to post and share both positive and negative material, it has resulted in a decrease in the effectiveness of conventional communication media (Mangold and Faulds, 2009). Web 2.0, often known as the social web, is currently popular among customers in general and tourists in particular (Šerić and Gil-Saura, 2012), owing to its low-cost and simple communication platforms (King et al., 2014).

To associate positive perceptions with tourist choices, tourism service providers must aim to maximize the impact of their communication initiatives (Godey et al., 2016). New response mechanisms for new situations must be developed to maximize the potential of social media-based interaction while minimizing potential negative consequences (Naumov and Tao, 2015). To achieve positive outcomes, tourism service providers must be aware of the importance of successfully handling both tourist-generated and firm-generated content through social platforms (Yunis et al., 2018). Recent research has also focused on the communication material produced by social networks (de Rosa et al., 2019). These two-way technologies aid new types of contact, including the products and services communication as well as the transfer of information *via* the Internet, thus influencing consumer views about brands and developing customer loyalty (Huerta-Álvarez et al., 2020).

### Tourism Service Provider and Tourist Generated Social Media Communication

Tourism service providers have several opportunities to build relationships and co-create value with clients through online social network communities (Kelly et al., 2010). The impact of firm-generated communication content will be determined by message sentiment, customer response, and customers’ inherent affinity for social media (Kumar et al., 2016).

Furthermore, from a business standpoint, the potential carried by travelers themselves *via* social media cannot be overlooked. There is an extensive literature assessing the influence of tourist-generated content and online word-of-mouth (eWOM) on final travel plans (Mauri and Minazzi, 2013). Several academics also contend that tourist-generated content has a substantial impact on how the image of tourism service providers is built (Stojanovic et al., 2018; Dedeoğlu et al., 2020). In short, tourists’ ability to spread positive messages and co-create value should not be underestimated.

### Value Co-creation

Customers’ bargaining power has grown, and they increasingly want to co-opt relationships with businesses – with many also wanting to add a customized touch to their encounters (Prahalad and Ramaswamy, 2004). According to Service-Dominant logic, the client is functionally entrenched into the service offering and thus accountable for any value added in the process (Ordanini and Pasini, 2008). When it comes to social media, there are light and heavy users. Lighter users are not very active in their co-creation activities (e.g., liking/commenting/feedback), while heavy users are significantly more likely to be enthusiastic about who they connect with and how they interact with them.

Differentiating oneself as a service provider may be best accomplished through social media. Because most service providers are almost homogenous (Yoganathan et al., 2015), therefore engagement and co-creation behaviors may help service provider to achieve a competitive advantage. Customers today are active participants in co-creation process rather than being just passive receivers of service offerings (McColl-Kennedy et al., 2017). As a result, the experiential needs of tourists can be addressed with the help of social media.

Co-creation on social media may involve input on service improvements as well as participation in the form of “liking,” “commenting,” and “sharing” of sponsored content. When value is co-creation *via* social media, then this may promise long-term benefits to the firm rather than just short-term benefits (Cambra-Fierro et al., 2018). Tourists must engage and interact during discussions based on certain situations in order for co-creation to happen (Franklin and Marshall, 2019). In short, social media aligns with S-D logic in such a way that it allows for conversations and knowledge sharing between the parties (Abeza et al., 2020), resulting in positive online consumer experiences (Casper Ferm and Thaichon, 2021).

### Online Customer Experience

Customer experience is used to gain a competitive advantage that is tough to replicate. Web 2.0 elements, such as interactivity, customer-to-customer (C2C) online recommendations, online word of mouth, or user-generated content, improve the possibilities of service provider-to-customer interactions. Furthermore, hardware advancements, such as handheld devices that enable real-time information sharing, make this whole process more complex (Balasubraman et al., 2002).

Customer experience is frequently theorized as a psychological construct: cognitive and affective characteristics have been uncovered in several elements of customer experience (Edvardsson, 2005). The goals of today’s service providers are to provide consumers with unique experiences and to immerse them in close-to-original surroundings on the Internet. Customers are becoming more aware of the subjective and symbolic character of social media sites. Rather than simply purchasing items or services, they aspire to immerse themselves in experience environments (Carù and Cova, 2006).

We consider online customer experience to be a psychological condition manifested as a subjective response to a web portal. On a consistent basis, the two psychological variables cognition and affect have been proven as prominent components of customer experience and customer behavior (Tynan and McKechnie, 2009). Because of the nature of Web 2.0 technology, virtual environments now exist in which the customer and firm collaborate to co-create valuable experiences (Kohler et al., 2011a).

Given the expansion of online communication platforms, online customer experience is an important topic for tourism service providers (Rose et al., 2011). On social media, online users are exposed to incoming sensory data from a variety of stimuli such as visual images, text-based content, audio, or visual delivery. The customer processes incoming sensory information from social media sites cognitively and affectively, resulting in the construction of an imprint in memory (Rose et al., 2012; Micu et al., 2019).

#### Cognitive and Affective Experiential State

Cognition has been found to be influenced by an individual’s emotional state in a marketing situation (Bagozzi et al., 1999). Following Gentile et al. (2007) cognitive experiential state is defined as “the component of online customer experience connected with thinking or conscious mental processes” (Rose et al., 2012).

According to Rose et al. (2012), the cognitive experiencial state is a state of flow. Flow is defined by Csikszentmihalyi (2000) as “the state in which people are so completely absorbed in an activity that nothing else seems to matter, the experience itself is so delightful that people will do it at tremendous cost, just for the sake of doing it.” A high level of concentration, control, challenge, enjoyment, and curiosity characterizes a state of flow (Hoffman and Novak, 2009). A consumer in a state of flow has a sense of satisfaction, confidence, and a desire to explore (Volle and Charfi, 2011).

Furthermore, data suggests that affective experiential state has an impact on judgments and decision-making. Affective experiential state has been defined as “the component of online consumer experience that engages one’s emotional system through the formation of moods, feelings, and emotions” (Gentile et al., 2007). Affective online consumer experiences are increasingly being recognized as major performance drivers in social media sites (Davidson and Vaast, 2010). Rose et al. (2012) linked the affective state of the experience to the state of mind, revealing that the emotions and feelings elicited during online engagement may have an impact on customer wellbeing.

### Customer Well-Being

All conceptualizations and metrics of customer wellbeing are based on the implicit or explicit assumption that higher levels of customer wellbeing result in higher levels of consumer quality of life. The Internet wellbeing metric was developed on the theoretical premise that users’ perceptions of the overall impact of the Internet are determined by their perceptions of the impact of the Internet in various life domains such as the marketplace, work life, leisure life, social life, education, community, sensual life, and so on. In turn, views of the Internet’s impact in a specific life domain (for example, work life) are influenced by perceptions of the Internet’s benefits and costs within that domain.

In general, based on the two motivational orientations (utilitarian and hedonic), customers’ perceived utility and enjoyment during website visits lead to an improvement in their quality of life in connected life domains (Grzeskowiak and Sirgy, 2007). Wellbeing is defined as “the consumer’s perception of the amount to which a brand (a consumer good or service) positively contributes to multiple life domains, resulting in an overall perception of the brand’s quality-of-life influence” (Grzeskowiak and Sirgy, 2007). Previous studies, in particular, have given evidence associating the value created when customers experience a service/product with wellbeing in the context of online experience (Bilgihan et al., 2015; Kim et al., 2016, 2019).

There are two key ramifications for tourism service providers. Firstly, tourist service providers might be claimed to function solely as a site for transactions (e.g., renting a room, purchasing a vacation package, etc.), i.e., as a platform for online shopping (Dedeke, 2016). Secondly, tourism service providers, can offer multiple points of interactive communication, as indicated by Bilgihan et al. (2016). Importantly, Kim and Kim (2017) demonstrated that consumers’ travel needs can be met prior to the actual trip, confirming the association between Internet use and wellbeing. Throughout this process, people not only search for information online, but also engage in social interactions that generate hedonic aspects online (Kim et al., 2019).

### Hypothesis Development

#### Tourism Service Provider Generated Social Media Communication and Value Co-creation

Firm-generated social media content has already been widely researched and tested with numerous predictor and outcome variables in diverse contexts. Researchers explored how firms build focused engagement objects through postings to their social media community members and how these individuals connect with these posts in ways that potentially co-create value in a study (Sorensen et al., 2017). In other studies, the impact of firm-generated content (FGC) on consumer brand awareness, brand loyalty, electronic word of mouth (Poulis et al., 2019), brand image (Bai and Yan, 2020), consumer digital engagement, and firm sales performance (Cheng et al., 2021), and customer purchase intention (Santiago et al., 2022) was investigated. Also, Cambra-Fierro et al. (2022) investigated the relationships between DMO (Destination Management Organization)-generated and tourist-generated communication and destination awareness, imagery, and perceived health safety. However, to the best of the authors’ knowledge, literature on tourism service providers’ generated social media content and its impact on value co-creation is still lacking. Therefore, we hypothesize that:

H_1_: Tourism Service Provider (TSP) generated social media communication positively influence value co-creation

#### Tourist Generated Social Media Communication and Value Co-creation

Füller (2010) studied what consumers expect from virtual co-creation and how consumers’ motives and personalities influence such expectations. According to the study’s findings, differently motivated consumer groups may have varying expectations of co-creation in terms of the process, co-creation content, and co-creation partners. Companies all over the world are starting to take social media seriously, seeing it as an essential component of their integrated marketing communication strategy. Because the global market is customer-focused and customers are embracing various social media platforms, it is critical to delight customers through co-creation, with execution taking place through social media (Yadav et al., 2016). In this regard, Yang and Li (2016) expanded co-creation theory by investigating the popularity of consumer-generated material, specifically the total number of comments on a post, which indicates knowledge exchange and sharing among consumers. Tourists do not always receive entire information about a service, experience, or destination from service providers, and may not even want it. As a result, individuals search online for complimentary information from numerous or a single source. Social networking platforms are becoming popular information repositories where travelers may publish and search for information on tourist experiences and co-create value (Borges-Tiago et al., 2019). Firms can boost customer commitment by encouraging customer and lead user participation in online communities (Rashid et al., 2019). Tourist co-creation of value through social networking sites is an element that destinations should consider as part of their strategy when a tourism service provider wants to govern its image and appeal (Bourliataux-Lajoinie et al., 2019). Further to this, Lam et al. (2020) stated that user-generated platforms can contribute to the process of online co-creation. Therefore, we posit that:

H_2_: Tourist generated social media communication positively influence value co-creation

#### Value Co-creation and Online Customer Experience

An open two-way communication is provided by social media networks through which mutually beneficial customer experiences can be designed and developed (Klaus, 2014). In a study, Zhang et al. (2018) emphasized that value co-creation in online platforms may result in better experiences for everyone. Firm-customer or customer–customer value co-creation is the value which is socially formed in an interaction-rich service setting. A study emphasized the significance of customer-customer interaction in a service setting and how it may add value to consumers’ experiences (Pandey and Kumar, 2020). Given consumers’ growing engagement in co-creating their online service experience, Pentina et al. (2021) proposed testing a multidimensional concept of online service experience co-creation. Furthermore, successful value co-creation is about more than just initiating communication; it is about communicating effectively in the sense that the service provider can understand customers’ true needs and meet their expectations (Lei et al., 2021) as well as improve their cognitive and affective experiential states (Anshu et al., 2021). Therefore, we hypothesize that:

H_3_: Value co-creation positively influence cognitive experiential state

H_4_: Value co-creation positively influence affective experiential state

#### Online Customer Experience and Customer Wellbeing

A captivating experience increases participants’ objective to live more sustainably (Kohler et al., 2011b). The researchers went on to say that individuals’ real-life behavior and attitudes are influenced by their behavior and actions in virtual worlds. A holiday, according to Baccarani and Cassia (2017), is articulated and occurs in four stages: (1) “choosing a destination and designing a trip”; (2) “traveling to the destination”; (3) “staying at a destination”; and (4) “transferring to another place or returning home.” The first stage (i.e., “choosing a destination and designing a trip”) represents the formation of tourists’ experiential expectations. Customers fantasize and anticipate their vacations during the first stage, as the anticipation of pleasure is a pleasure in and of itself, boosting their wellbeing. However, a limited number of studies have shown that customer experiences and the quality of these experiences should promote consumer wellbeing (Baccarani and Cassia, 2017). Kim et al. (2019) found that hedonic value is a greater predictor of wellbeing than functional value in a travel website, despite the fact that both values are successful in promoting wellbeing. Travel websites may elicit cognitive and affective experiences, leading to increased wellbeing. Similarly, it was highlighted in another study that experience memory positively influenced emotional wellbeing, which included customers’ contentment, enjoyment, and pleasant sensations (Baloglu et al., 2019). Also, Xu et al. (2021) postulated that online customer experience in virtual travel communities has a direct impact on customers’ social wellbeing. Therefore, we hypothesize that:

H_5_: Cognitive experiential state positively influences customer wellbeing

H_6_: Affective experiential state positively influences customer wellbeing

### Theoretical Framework

**FIGURE 1:**
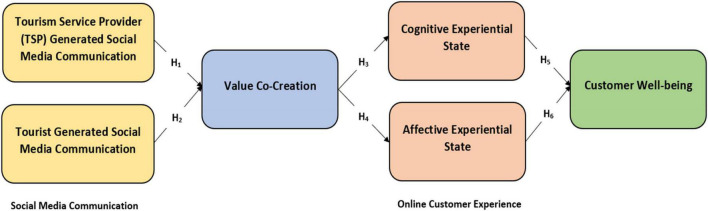
Proposed framework.

The framework proposed in Figure 1 is primarily based on two theories, i.e., Uses and Gratification theory and Horizontal Spillover theory.

Uses and gratification theory can be used to better understand why people participate in co-creation on online platforms. According to this notion, users are likely to benefit from their participation in social media (Rashid et al., 2019). This idea is based on two assumptions about media users. Firstly, it portrays media consumers as actively choosing the media they consume. From this point, we may presume that customers are engaged and motivated in their media choices, which will make the co-creation process easier. Second, people are aware of their motivations for choosing various media options, and they rely on this knowledge to make media choices that will help them achieve their specific wants and needs. In our instance, this refers to existing and potential tourists who are looking for information on social media in order to make trip plans. Furthermore, the Uses and Gratifications paradigm emphasizes the individual’s power over the power of the media. Individual differences act as a buffer between media and their consequences. As a result, even if everyone gets the same media message, they will not all be affected in the same manner. This also applies to the setting of this study, where social media communication generated by tourism service providers and tourists is more powerful than the social media platform itself. Furthermore, each customer may perceive the value co-creation process differently, resulting in various online experiences and increased/decreased customer wellbeing.

Going forward, the horizontal spillover method can be used to improve subjective wellbeing. According to Horizontal Spillover theory, contentment or discontent in one domain has an effect on a surrounding domain. There is a lot of evidence in the quality-of-life literature that suggests affect in one life domain influences affect in another that isn’t superordinate or subordinate to it but is on the same plane in the general hierarchy of life domains and concerns (Sirgy, 2012). Because the current study attempts to analyze the impact of social media communication on consumer wellbeing *via* the mediation of value co-creation and online experience, this theory lends itself nicely to our suggested framework. If social media communication helps the value co-creation process, it may result in a positive online experience, which may result in increased customer wellbeing. On the other hand, if social media communication fails to co-create value, it may result in a negative online experience and diminished customer wellbeing.

## Materials and Methods

For this study, Malaysia has been selected as a geographical focus. Malaysia is one of the southeast Asian countries that has seen tremendous expansion in the tourism industry in recent years (Hirschmann, 2020). Also, tourism is the third-largest contributor to Malaysia’s GDP, accounting for 5.9% of the country’s overall GDP in 2018 (Hirschmann, 2020). In order to investigate the proposed relationships, an online survey form was formulated. To circulate the questionnaire to the respondents, several social media pages of tourism service providers and tourism social media groups were identified. The link to the online questionnaire was then posted over the identified social media platforms (Facebook, Instagram, and Twitter in our case), and also shared through direct messages with some group admins/members who were actively liking, commenting, and sharing the content. The data was collected over the span of 4 months i.e., from October 2021 to January 2022, as this was the time when COVID-19 restrictions were relaxed in the Malaysia and domestic tourism was allowed for the citizens. Therefore, customers were actively engaged in making their travel plans. A seven-point likert scale was used as a basis for the scale. For tourism service provider (TSP)- and tourist generated social media communication, the scale were adapted from Huerta-Álvarez et al. (2020). The scale for value co-creation was adapted from Casper Ferm and Thaichon (2021). Furthermore, for cognitive and affective experiential states, the scale was adapted from Rose et al. (2012). Whereas, the scale for customer wellbeing was adapted from Kim et al. (2019). As a result, the authors were able to collect 361 valid responses from the Malaysian citizens. For the analysis of the data, Smart PLS software was used.

## Results

### Confirmatory Factor Analysis, Measurement, and Structural Models

Our hypothesized model exhibited a satisfactory model fit against the chosen fit indices i.e., *P*-Value ≥ 0.05, RMSEA = 0.006, GFI = 0.93, NFI = 0.941, AGFI = 0.918, CFI = 0.999, TLI = 0.999, CMIN (Chisq/df) = 1.012 for the overall measurement model, as all of such measures are predefined through earlier literature. Our findings also reported the accepted values for the overall structural model i.e., *P*-Value ≥ 0.05, RMSEA = 0.021, GFI = 0.918, NFI = 0.931, AGFI = 0.906, CFI = 0.990, TLI = 0.989, and CMIN (Chisq/df) = 1.161. Further, all items were statistically significant with sufficient amount of factor loading to their respective factor (*P* < 0.01). As shown in Table 1, factor loading for Tourism service provider communication (TSP) ranged between 0.83–0.86, Tourist generated social media communication (TGSM) 0.88–0.89, Affective experiential state (AES), 0.84–0.85, Cognitive experiential state (CES) 0.86–0.89, Value co-creation (VCC) 0.715–0.724, and lastly for the Customer wellbeing (CWB) ranged between 0.90–0.92.

**TABLE 1:** Standardized factor loadings.

Variables	Item	TSP	TGSM	VCC	CES	AES	CWB
Tourism Service Provider Generated Social Media Communication	I’m satisfied with communication generated by the tourism service provider on social media.	0.838					
	The level of communication on social media and other technologies from the tourism service provider meets my expectations.	0.834					
	Communication on social media from the tourism service provider is very attractive.	0.857					
	Compared to social media communication from other tourism service providers, communication generated by this tourism service provider is effective.	0.863					
Tourist Generated Social Media Communication	I’m satisfied with communication generated by other tourists on social media about the tourism service provider.		0.892				
	The content generated by other tourists about the tourism service provider on social media is very attractive.		0.881				
	The content generated by other tourists about the tourism service provider on social media provides me with different ideas about this tourism service provider.		0.895				
	The content generated by other tourists about the tourism service provider on social media helps me formulate ideas about this tourism service provider.		0.883				
Value Co-creation	I often check the tourism service provider social media to get feedback from other customers.			0.731			
	In the tourism service provider social media, I usually offer my suggestions for the improvement of customer service and/or tourism services.			0.73			
	If I am unhappy over one of my tourism experiences, I will make a suggestion for improvement on the tourism service provider’s social media channels.			0.715			
	I enjoy liking posts from the tourism service provider on social media.			0.724			
	I regularly like posts from the tourism service provider on social media.			0.73			
	Liking posts from the tourism service provider is something that I do often while on social media.			0.723			
	I enjoy commenting on posts from the tourism service provider on social media.			0.715			
	I regularly comment on posts from the tourism service provider on social media.			0.726			
	Commenting on posts from the tourism service provider is something that I do often while on social media.			0.725			
Cognitive Experiential State	Do you think you have ever experienced “flow” on the social media?				0.865		
	In general, how frequently would you say you have experienced “flow” when you use the social media?				0.869		
	Most of the time I use the social media I feel that I am in “flow.”				0.891		
Affective Experiential State	Pleased					0.842	
	Unhappy					0.846	
	Relaxed					0.856	
	Excited					0.858	
	Annoyed					0.854	
	Guided					0.847	
	Autonomous					0.84	
	Influenced					0.847	
Customer Wellbeing	The social media communication met my overall need for wellbeing.						0.905
	The social media communication played a very important role in my social wellbeing.						0.906
	The social media communication played an important role in my travel wellbeing.						0.924
	The social media communication played an important role in enhancing my quality of life.						0.915

### Reliability and Validity Analysis

The overall reliability of the scale being utilized for this study was evaluated through the internal consistency of each construct’s items. Stated by Nunnally (1978), values of already developed scales items greater than 0.70 are considered to be acceptable, hence the reliability of the scale will be achieved. In our case, the Cronbach α coefficients of all constructs were; Tourism service provider communication (TSP) 0.87, Tourist generated social media communication (TGSM) 0.911, Affective experiential state (AES) 0.945, Cognitive experiential state (CES) 0.847, Value co-creation (VCC) 0.887, and lastly for the Customer wellbeing (CWB) 0.933, therefore our scale exhibited strong psychometric properties with internal consistency. Further, to assess the convergent validity of the scale, measures such as; Average variance extracted (AVE), Standardized Factor loading (SFL), and composite reliability were utilized. Values of CR > 0.70, AVE > 0.50, and SFL > 0.60 show the acceptability and feasibility of the findings (Bagozzi and Yi, 1988). Based on our findings, all of the aforementioned criteria were met, hence displayed in Table 2.

**TABLE 2:** Convergent validity and reliability.

	Cronbach’s Alpha	rho_A	Composite reliability	Average variance extracted (AVE)
AES	0.945	0.947	0.954	0.721
CES	0.847	0.85	0.907	0.766
CWB	0.933	0.935	0.952	0.832
TGSM	0.911	0.911	0.937	0.789
TSP	0.87	0.871	0.911	0.719
VCC	0.887	0.887	0.909	0.525

*AES, affective experiential, CES, cognitive experiential state, CWB, customer wellbeing, TGSM, tourist generated social media communication, TSP, tourism service provider communication, VCC, value co-creation.*

Furthermore, in order to assess the discriminant validity of the scale being utilized, we compared the correlation coefficient of all research construct with the square roof their average variance extracted (AVE) (Bagozzi and Yi, 1988), and based on the outcomes, the discriminant validity was attained (see Table 3). For discriminant validity, the correlation coefficient of all variables was compared with the square root of average variance extracted (AVE) (Bagozzi and Yi, 1988), and the discriminant value was achieved accordingly (Table 3).

**TABLE 3:** Discriminant validity.

	CR	AVE	MSV	MaxR (H)	TSP	TGM	VCC	CES	AES	CWB
TSP	0.91	0.72	0.424	0.872	**0.792**					
TGM	0.94	0.79	0.229	0.911	0.455***	**0.847**				
VCC	0.90	0.53	0.424	0.887	0.651***	0.478***	**0.682**			
CES	0.90	0.77	0.255	0.849	0.505***	0.298***	0.453***	**0.806**		
AES	0.95	0.72	0.19	0.945	0.401***	0.163**	0.321***	0.436***	**0.825**	
CWB	0.95	0.83	0.111	0.934	0.071	–0.02	0.184**	0.333***	0.319***	**0.88**

**p < 0.010, ***p < 0.001.

*AES, affective experiential, CES, cognitive experiential state, CWB, customer wellbeing, TGSM, tourist generated social media communication, TSP, tourism service provider communication, VCC, value co-creation.*

Moreover, Kock et al. (2012) introduced the full collinearity test as a thorough approach for assessing both vertical and lateral collinearity at the same time. This approach generates variance inflation factors (VIFs) for all latent variables in a model. A VIF greater than 3.3 is recommended as an indication of pathological collinearity, as well as indicating a model may be tainted by common technique bias. As a result, if all VIFs from a comprehensive collinearity test are equal to or less than 3.3, the model is free of common method bias. Table 4 displays the VIFs derived from a thorough collinearity test for all of the latent variables in our model.

**TABLE 4:** Test for common method bias (Inner VIF).

	TSP	TGM	VCC	CES	AES	CWB
TSP	–	–	1.198	–	–	–
TGM	–	–	1.198	–	–	–
VCC	–	–	–	1	1	–
CES	–	–	–	–		1.179
AES	–	–	–	–	–	1.179
CWB	–	–	–	–	–	–

Lastly, Demographic data of the research is also shown in Table 5, which shows the maximum coverage possible for this study.

**TABLE 5:** Demographics of the research.

Demographic Variable	Range	Number of respondents
Age	18–25	95
	26–35	105
	36–45	50
	46–55	66
	56+	45
Gender	Male	276
	Female	85
Ethnicity	Malay	193
	Chinese	109
	Indian	45
	Others	14
Social media usage	Heavy user	169
	Moderate user	122
	Light user	70
Type of Tourism service provider with whom social media communication recently experienced	Hotel/resort	57
	Transportation	36
	Tourism Attraction/destination	76
	Adventure and Recreation	86
	Events/conference/expo	41
	Others	65

### Hypotheses Testing

We tested the hypothesized model (Figure 1) *via* the structural equation modeling (SEM) technique. Based on the findings, our hypothesized model reported an adequate amount of variance in the dependent variables i.e., Affective experiential state (AES) 8.5%, Cognitive experiential state (CES) 15.2%, Value co-creation (VCC) 0.375, and lastly 12.4% for the Customer wellbeing (CWB) through their predictors. Referring to the individual hypothesis outcomes, hypothesis-1 Tourism service provider communication (TSP) → Value Co-creation was accepted (β = 0.476, *p* < 0.001), hence meeting the research expectations. Hypothesis-2 i.e., Tourist generated social media communication (TGSM) → Value Co-creation was also accepted based on its positive path coefficient and significant P-value (β = 0.237, *p* < 0.001), therefore again meeting the predefined research assumptions. Hypothesis 3 and 4 assessing the impact of Value Co-creation on both Cognitive experiential state (CES) and Affective experiential state (AES) were also accepted based on their significant statistical values i.e., VCC→CES (β = 0.393, *p* < 0.001), VCC→AES (β = 0.293, *p* < 0.001). Lastly, the effect of both Cognitive experiential state (CES) and Affective experiential state (AES) was tested on Customer wellbeing, hence hypotheses 5 and 6 both were accepted with statistical values such as CES→CWB (β = 0.214 *p* > 0.001), and AES→CWB (β = 0.218 *p* > 0.001). Path coefficients of the research framework are displayed in Table 6.

**TABLE 6:** Path coefficients of the proposed model.

	Original sample (O)	Sample mean (M)	Standard deviation (STDEV)	T statistics (| O/STDEV|)	*P*-Values
TSP –> VCC	0.476	0.477	0.049	9.776	0
TGSM –>VCC	0.237	0.238	0.071	3.361	0.001
VCC –>CES	0.393	0.394	0.048	8.174	0
VCC –>AES	0.296	0.3	0.045	6.64	0
CES –>CWB	0.214	0.217	0.042	5.143	0
AES –>CWB	0.218	0.221	0.043	5.104	0
					

Referring to the mediation analysis which was conducted using bootstrapping technique with sample size of 5000 and at 95% confidence interval, it was found that Value co-creation (VCC) partially mediated between the Tourism service provider generated social media communication (TSP), Tourist generated social media communication (TGSM), and both Cognitive experiential state (CES) and Affective experiential state (AES). It was also observed that both Cognitive experiential state (CES) and Affective experiential state (AES) partially mediated between Value Co-creation (VCC) and Customer wellbeing (CWB). The result of the mediation analysis is shown in Table 7.

**TABLE 7:** Mediation analysis results.

	Original sample (O)	Sample mean (M)	Standard deviation (STDEV)	T Statistics	*P*-Values
TGSM –>VCC –>CES –>CWB	0.02	0.02	0.008	2.456	0.014
TSP –>VCC –>CES –>CWB	0.04	0.041	0.01	3.935	0
TGSM –>VCC –>AES	0.07	0.072	0.026	2.739	0.006
TGSM –> VCC –>CES	0.093	0.094	0.032	2.88	0.004
TSP –>VCC –>CES	0.187	0.188	0.03	6.221	0
VCC –>AES –>CWB	0.064	0.066	0.014	4.679	0.064
TGSM –>VCC –>AES –>CWB	0.015	0.016	0.006	2.476	0.015
TSP –>VCC –>AES –>CWB	0.031	0.031	0.007	4.355	0.031
VCC –>CES –>CWB	0.084	0.085	0.019	4.317	0.084
TSP –>VCC –>AES	0.141	0.143	0.025	5.733	0.141

## Discussion

The aim of the study was to assess the impact of tourism service provider (TSP) and tourist generated social media communication on value co-creation, online customer experience (which comprised of cognitive and affective experiential state), and customer wellbeing. Firstly, the authors assumed that tourism service provider (TSP) and tourist generated social media communication exert a positive influence on value co-creation process. Our findings supported this assumption which is in harmony with the existing literature as well (Mauri and Minazzi, 2013; Dedeoğlu et al., 2020). Secondly, the authors examined the relationship between value co-creation process and online customer experience. The research findings supported this argument as well, hence proving that value co-creation positively influence the online customer experience (cognitive and affective experiential states). These findings are also well aligned with the existing literature (Abeza et al., 2020; Casper Ferm and Thaichon, 2021). Thirdly, the study posited that online customer experience have a positive influence on customer wellbeing which was also accepted as per the study’s findings. If customers enjoy a favorable and positive online experience then it will consequently alleviate their wellbeing (Kim and Kim, 2017). Fourthly, this study also investigated the mediation effects of value co-creation between social media communication and online customer experience. Also, the mediating role of online customer experience between value co-creation and customer wellbeing was assessed. The mediation results showed that all the mediators partially mediated the relationship between predictor and outcome variables.

### Theoretical Implications

This study has tried to establish a theoretical relationship between all some noteworthy variables (social media communication, value co-creation, online customer experience, and customer wellbeing) in order to better understand the phenomenon and its antecedents/outcomes. Our study findings reveal that the more tourism service providers are prepared to effectively manage the social media content (either service provider-generated or tourist-generated), greater will be chances for co-creation of value between the service provider and tourists/customers. Value co-creation in turn allows customers to interact and engage in the activities offered by the service providers. In social media platforms as well, if such opportunities are given to the customers, then it is very likely that their online experience will be pleasurable. Also, value co-creation may play a vital role in enhancing and improving customers’ cognitive and affective experiential states. Furthermore, online customer experience plays a significant role in shaping customer’s/tourist’s perception and enhancing their wellbeing even before they have a physical encounter with a service provider. Also, the mediation results highlighted that the mediating variables partially mediate the relationships between predictor and outcome variables, and the intervention of these variables will further strengthen these theoretical relationships. Moreover, the current study advanced the existing theoretical knowledge by expanding Uses and Gratification theory and Horizontal Spill-over theory in the tourism service context. The research findings elaborated that for customers who are looking for information on social media in order to make trip plans, the social media communication generated by tourism service providers and tourists is more powerful than the social media platform itself. Furthermore, each customer may perceive the value co-creation process differently, resulting in varying online experiences and different levels of customer wellbeing. Also, referring to Horizontal Spill-over theory, if social media communication facilitates the value co-creation process, it may result in a positive online experience, which may result in enhanced customer wellbeing causing the spill-over from one domain to another.

### Practical Implications

The findings drawn from this study will benefit the practitioners in the tourism industry at large. The practitioners will realize the importance of social media communication and the role it plays in constructing the image of the service providers. The tourism marketers must realize that in today’s digital age, it is obligatory to effectively manage firm-generated and user-generated content on their social media platforms as this will not only carry short-term benefits but would help in long-term sustainability as well. The tourism service providers should note that the content that is generated for social media campaigns must be two-way so that the current and potential customers get a chance to interact and engage with the firm.

## Data Availability Statement

The raw data supporting the conclusions of this article will be made available by the authors, without undue reservation.

## Author Contributions

MA: idea inception and write-up. AS: manuscript review and proofreading. MS: data analysis and write-up. All authors contributed to the article and approved the submitted version.

## Conflict of Interest

The authors declare that the research was conducted in the absence of any commercial or financial relationships that could be construed as a potential conflict of interest.

## Publisher’s Note

All claims expressed in this article are solely those of the authors and do not necessarily represent those of their affiliated organizations, or those of the publisher, the editors and the reviewers. Any product that may be evaluated in this article, or claim that may be made by its manufacturer, is not guaranteed or endorsed by the publisher.
